# Whole-genome sequencing of SARS-CoV-2 reveals the detection of G614 variant in Pakistan

**DOI:** 10.1371/journal.pone.0248371

**Published:** 2021-03-23

**Authors:** Massab Umair, Aamer Ikram, Muhammad Salman, Adnan Khurshid, Masroor Alam, Nazish Badar, Rana Suleman, Faheem Tahir, Salmaan Sharif, Joel Montgomery, Shannon Whitmer, John Klena

**Affiliations:** 1 National Institute of Health, Islamabad, Pakistan; 2 Centers for Disease Control and Prevention, Atlanta, Georgia, United States of America; Defense Threat Reduction Agency, UNITED STATES

## Abstract

Since its emergence in China, severe acute respiratory syndrome coronavirus 2 (SARS-CoV-2) has spread worldwide including Pakistan. During the pandemic, whole genome sequencing has played an important role in understanding the evolution and genomic diversity of SARS-CoV-2. Although an unprecedented number of SARS-CoV-2 full genomes have been submitted in GISAID and NCBI, data from Pakistan is scarce. We report the sequencing, genomic characterization, and phylogenetic analysis of five SARS-CoV-2 strains isolated from patients in Pakistan. The oropharyngeal swabs of patients that were confirmed positive for SARS-CoV-2 through real-time RT-PCR at National Institute of Health, Pakistan, were selected for whole-genome sequencing. Sequencing was performed using NEBNext Ultra II Directional RNA Library Prep kit for Illumina (NEW ENGLAND BioLabs Inc., MA, US) and Illumina iSeq 100 instrument (Illumina, San Diego, US). Based on whole-genome analysis, three Pakistani SARS-CoV-2 strains clustered into the 20A (GH) clade along with the strains from Oman, Slovakia, United States, and Pakistani strain EPI_ISL_513925. The two 19B (S)-clade strains were closely related to viruses from India and Oman. Overall, twenty-nine amino acid mutations were detected in the current study genome sequences, including fifteen missense and four novel mutations. Notably, we have found a D614G (aspartic acid to glycine) mutation in spike protein of the sequences from the GH clade. The G614 variant carrying the characteristic D614G mutation has been shown to be more infectious that lead to its rapid spread worldwide. This report highlights the detection of GH and S clade strains and G614 variant from Pakistan warranting large-scale whole-genome sequencing of strains prevalent in different regions to understand virus evolution and to explore their genetic diversity.

## Introduction

Since its emergence in Hubei province, China, in December 2019, the highly contagious severe acute respiratory syndrome coronavirus 2 (SARS-CoV-2) has spread across the world, causing more than 70 million cases and 1.6 million deaths till December 13, 2020 [1]. The first case of SARS-CoV-2 infection in Pakistan was reported on February 26, 2020 when a traveler returned from Iran tested positive for the virus [2]. Sustained local transmission has resulted in 432,327 cases and 8,653 deaths in the country as of December 10, 2020 (http://covid.gov.pk/stats/pakistan).

The virus is a member of *Coronaviridae* family, genus *Betacoronavirus*, and has a positive-sense single-stranded RNA genome. The genome is about 30kb and encodes sixteen nonstructural proteins (NSP1-NSP16) and four structural proteins (nucleocapsid, envelop, membrane, and spike glycoprotein). The spike (S) protein is involved in SARS-CoV-2 entry into host cells through binding with angiotensin-converting enzyme 2 (ACE2) receptor. The receptor binding domain (RBD) in the S protein interacts with ACE2 receptor that eventually leads to the fusion of virus to host cell membranes [3]. Recently, the detection of a D614G mutation in the S protein of SARS-CoV-2 and subsequent global spread has received tremendous attention. The D614G mutation has been shown to be present in the S2 domain of spike protein and is critical for cleavage of S1 facilitating the fusion of virus with host cell membrane [4, 5]. However, the role of D614G mutation in the emergence of a more transmissible variant of SARS-CoV-2 was first highlighted by Korber et al. through the development of an analysis pipeline [4]. Since the initial report, several studies on the role of D614G amino acid change in enhanced infectivity of SARS-CoV-2 have been documented [6–8]. The G614 variant which arose in Europe during February 2020, rapidly spread to other parts of the world occurring in over 74% of all published sequences by June 2020 [9].

As per GISAID nomenclature, the SARS-CoV-2 sequences have been grouped into 6 major clades i.e. L (the clade harboring the Wuhan reference strain), G, GH, GR, S, & V. Genomic epidemiology of SARS-CoV-2 has shown that L and S clades emerged during the early phase of pandemic (January-February) and rapidly declined afterward. In Asia, the L clade was found at a frequency of 48% in January and 2% in July whereas the S clade which was commonly circulating in January (31%), almost disappeared after August. Unlike the L and S clades, the G clade sequences were reported at low frequency during January-February 2020 and progressively increased with a peak in June (25%), following with constant detection till December 2020 (17%). The G clade along with GH and GR started emerging on a global scale during March 2020. The GR clade was found at the highest frequency (49%) in September 2020 and GH (45%) in December 2020 (https://www.gisaid.org/epiflu-applications/phylodynamics/).

In case of emerging viruses, genomic epidemiology has proven to be a useful tool for investigating the outbreak and tracking virus evolution and spread [10–12]. During SARS-CoV-2 pandemic, genomic epidemiology has been used to track the nature of transmission of virus in several countries and for investigating the introduction of multiple importations [13–20]. To understand SARS-CoV-2 evolution and genetic diversity of circulating strains, transmission patterns, and detection of variants such as G614, whole-genome sequencing is imperative. Although more than 150,000 SARS-CoV-2 genome sequences have been deposited in the GISAID as of December 02, 2020, data from Pakistan is limited (n = 10) (https://www.gisaid.org/). We hereby report the complete genome sequences and phylogenetic analysis of five SARS-CoV-2 strains detected in Pakistan.

## Materials and methods

### Sample selection

The oropharyngeal swabs of five patients that were confirmed positive for SARS-CoV-2 through real-time RT-PCR using commercially available kit genesig® Real-Time PCR Coronavirus COVID-19 (Primerdesign Ltd., UK) at the Department of Virology, National Institute of Health, Pakistan, were selected for whole-genome sequencing. The rationale for the selection of these samples was quality (RNA concentration of >10 ng/ul) and quantity (>1.5 ml volume) of the samples and low cycle threshold (Ct) values on real-time RT-PCR. Written informed consent was obtained from all study participants and the study design was approved by the Internal Review Board of the National Institute of Health (VIR-PHLD/IRB-2020/05).

### RNA extraction and next generation sequencing

The RNA was extracted from oropharyngeal swabs using the QIAamp Viral RNA Mini kit (Qiagen, Hilden, Germany) according to the manufacturer’s instructions. The NEBNext Ultra II Directional RNA Library Prep kit for Illumina (NEW ENGLAND BioLabs Inc., MA, US) was used for library preparation and whole-genome sequencing was performed on Illumina iSeq 100 instrument (Illumina, San Diego, US).

### Genome assembly

Genomes were assembled using in-house scripts which iteratively mapped reads (3x) with bwa-mem to the WIV04 (MN996528) reference and called the consensus genomes with Geneious (version 10.1.64, gaps filled with reference/intermediate sequences). Final consensus sequences were called with samtools mpileup (-A -d 6000000 -B -Q 0) and piping the output to iVar consensus (-m 2 -n N). Briefly, the samtools pileup command generates a pileup of alleles from the bam files using these parameters: include orphan read pairs (-A), set the maximum depth of reads at each position to 6000000 (-d 6000000), disable base alignment quality (BAQ) computation (-B), minimum base quality for mapping is 0 (-Q 0). The iVar consensus command was run with the following parameters: minimum quality (Default: 20), minimum frequency threshold (Default: 0.03), minimum depth to call a consensus (-m 2), and a character to call in regions with coverage lower than the specified minimum depth (-n N). Minor viral variants were assessed using bam files using the same parameters (samtools mpileup -A -d 6000000 -B -Q 0 piped to ivar variants (-q 20 (default) -t 0.03 (default) -m 2). The significance of minor viral variants was assessed using the Fisher Exact test in iVar to evaluate whether the frequency of the alternate allele is significantly higher than the mean error rate at that position.

### Phylogenetic analysis

All available SARS-CoV-2 sequences till December 7, 2020 were downloaded from GISAID. This dataset was filtered to remove sequences without metadata, genomes with greater than 1% of ambiguous bases (qiime genome-sampler filter-seqs—p-max-proportion-ambiguous 0.01), and partial genomes (qiime genome-sampler filter-seqs—p-min-length 29000—p-max-length 30001). To generate a subsampled, but comprehensive view of all available SARS-CoV-2 sequences the complete genomes dataset was further subsampled by longitudinal time (n = 183 genomes) (qiime genome-sampler sample-longitudinal default settings), closest diversity (n = 388 genomes) (qiime genome-sampler sample-diversity—p-percent-id 0.9990), and nearest neighbors (n = 7 genomes) to the new SARS-CoV-2 sequences under investigation (qiime genome-sampler sample-neighbors—p-percent-id 0.9992—p-samples-per-cluster 3). These subsampled datasets were combined with all available complete genomes (with less than 1% of ambiguous bases) from surrounding geographic regions: India, Iran, Oman, and available sequences from Pakistan (no complete genomes were available from Afghanistan, Tajikistan, the Xinjiang and Tibet provinces of China) (n = 3625 genomes). Genomes (n = 4176) were aligned with mafft/7.450 and phylogentic relationships were inferred using IQtree/1.6.8 with 1000 bootstrap iterations (-bb 1000 -m GTR+G4). Trees were visualized with the ggtree package from R. The whole genomes sequenced in the current study have been submitted to the GISAID and have the following accession ID’s: EPI_ISL_468159 to EPI_ISL_468163.

## Results

**Fig 1:**
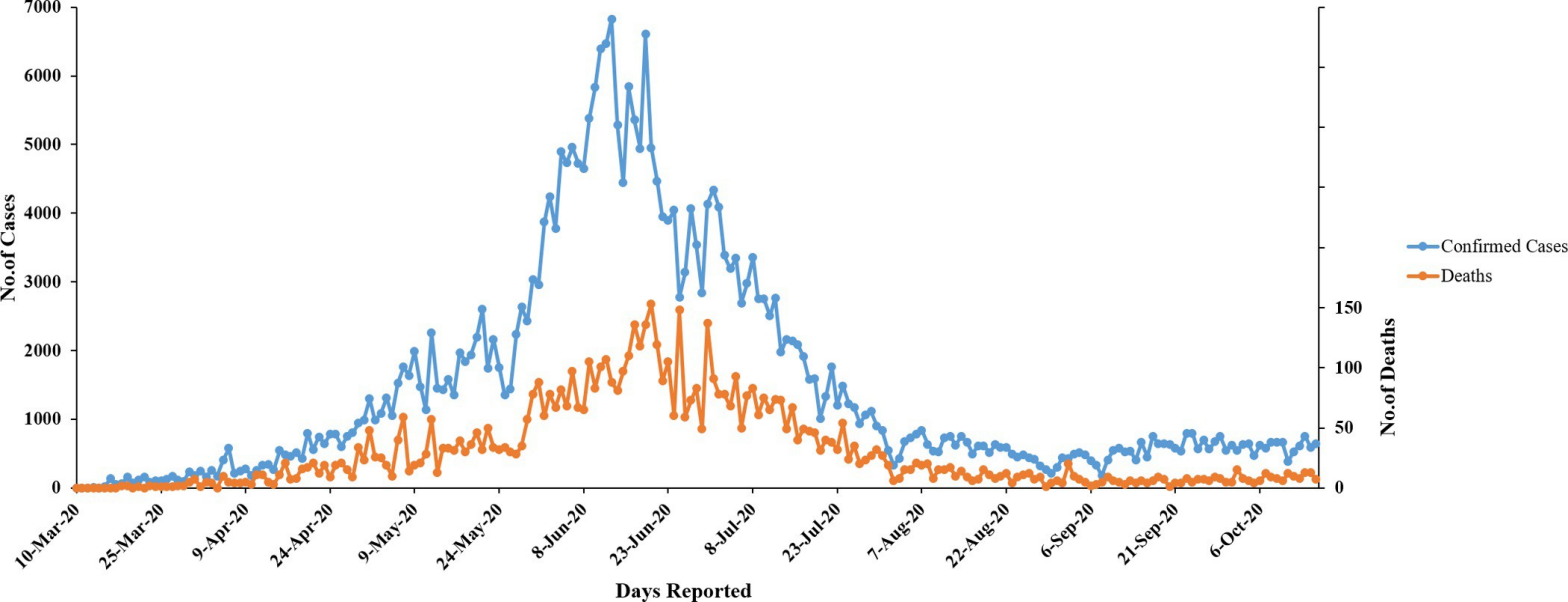
Daily reported COVID-19 cases and deaths in Pakistan since March 2020 to October 16, 2020. The data of confirmed positive cases and deaths were obtained from Government of Pakistan dashboard (https://covid.gov.pk/stats/pakistan).

In Pakistan, the first confirmed case of CVOID-19 was reported on 26^th^ February, 2020. Till March, a total of 2021 cases and 26 deaths were reported. The number of cases increased rapidly in April (n = 14,778) and May (n = 52,679). However, the peak of pandemic was witnessed in June with 141,010 cases. Since June, Pakistan saw a decline in COVID-19 cases (July n = 65,676; August n = 17,003; September n = 16,657 and October n = 21,164). The total deaths due to COVID-19 in Pakistan were n = 359 in April, n = 1184 in May, n = 2852 in June, n = 1575 in July, n = 328 in August, n = 186 in September, and n = 339 in October (Fig 1).

The clinical and demographic details of the patients enrolled in current study along with real-time PCR cycle threshold (Ct) values are shown in Table 1. These five oropharyngeal samples had the lowest Ct values among the available samples and full genome of SARS-CoV-2 was successfully obtained from these. The sequencing stats of Pakistani strains discussed in the current study are shown in Table 2.

**Table 1:** Clinical and demographic details of SARS-CoV-2 patients.

Lab ID	Date of collection	Age (years)	Gender	Sign & Symptoms	District/Location	Real-time PCR Cycle threshold (Ct) value
NIH-45090	02-June-2020	49	Female	Fever, cough & difficulty in breathing	Islamabad	14
NIH-45579	02-June-2020	46	Female	Fever & cough	Islamabad	12
NIH-45143	02-June-2020	55	Female	Fever & cough	Rawalpindi	14
NIH-44905	02-June-2020	87	Male	Fever & cough	Islamabad	15
NIH-HAS001	02-June-2020	23	Male	Fever & cough	Islamabad	16

**Table 2:** Sequencing stats of the five Pakistan strains.

Sequence Name	Average Coverage	% of Coverage compared to MN996528 Reference (# missing bases)
NIH-45090	213.95106	99.95 (12)
NIH-45579	86.9552	99.95 (12)
NIH-45143	21.85721	99.94 (17)
NIH-44905	15.90726	99.74 (75)
NIH-HAS001	19.84407	99.89 (31)

**Fig 2:**
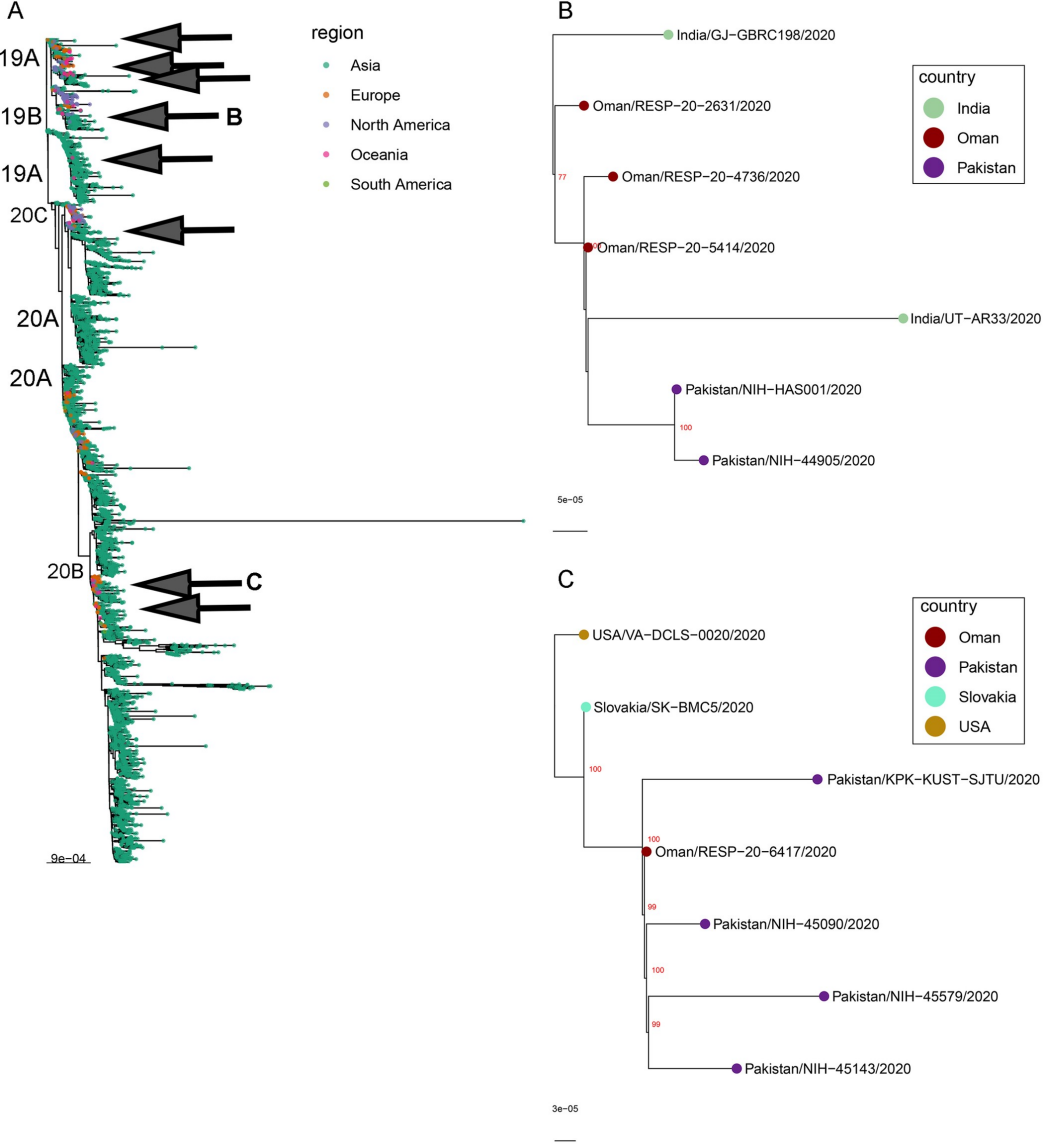
Inference of SARS-CoV-2 phylogenetic relationships. The phylogenetic tree was reconstructed using Maximum Likelihood method with bootstrap 1000 replicates (A). Globally subsampled SARS-CoV-2 genomes supplemented with additional complete genomes from countries surrounding Pakistan were included. Pakistani strains are highlighted with grey arrows. B) Expansion of clade containing sequences NIH-44905 and NIH-HAS001. C) Expansion of clade containing sequences NIH-45090, NIH-45579, and NIH-45143.

According to GISAID clade nomenclature (https://www.gisaid.org/references/statements-clarifications/clade-and-lineage-nomenclature-aids-in-genomic-epidemiology-of-active-hcov-19-viruses/), Pakistani strains NIH-44905 and NIH-HAS001 belong to S clade having characteristic genetic markers C8782T, T28144C, and NS8-L84S whereas strains NIH-45090, NIH-45143, and NIH-45579 belong to GH clade having marker variations C241T, C3037T, A23403G, G25563T, S-D614G, and NS3-Q57H. For further classification of SARS-CoV-2 strains detected in Pakistan, all available sequences were categorized into clades defined by the Nextstrain (https://docs.nextstrain.org/en/latest/tutorials/SARS-CoV-2/steps/naming_clades.html). The Nextstrain classification has grouped SARS-CoV-2 strains into five major global clades: 19A, 19B, 20A, 20B and 20C. Phylogenetic analysis based on the whole genomes showed that three strains NIH-45090, NIH-45143, and NIH-45579 belong to 20A clade and clustered with strains from Oman, Slovakia, United States and Pakistani strain EPI_ISL_513925. The other two strains (NIH-44905 and NIH-HAS001) grouped into 19B clade along with viruses from India and Oman (Fig 2). Based on analysis of available sequence data from Pakistan, we have observed 8 separate introductions of SARS-CoV-2 into Pakistan (the earliest sequence is from March 12, 2020). Most of these clades consist of a single sequence, except for the two clades presented here, and a third monophyletic clade from Lahore collected on November 5, 2020. Our analysis suggests multiple importations of SARS-CoV-2 into Pakistan and provides evidence of clustered outbreak/community transmission.

**Fig 3:**
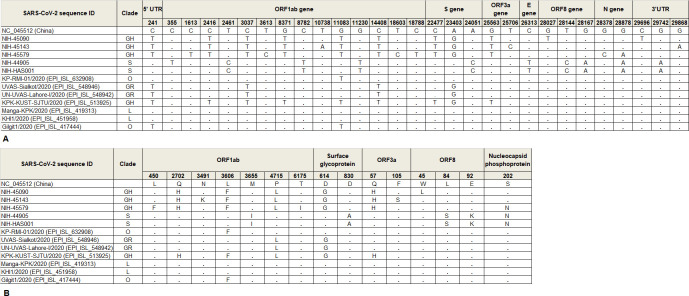
Comparison of SARS-CoV-2 isolates sequenced in the current study with the prototype strain and Pakistan isolates. The nucleotide changes are shown in A and the amino acid variations are presented in B.

We have analyzed the genome-wide single-nucleotide polymorphisms (SNPs) and found nine common nucleotide variations (241C>T, 2416C>T, 3037C>T, 8371G>T, 11083G>T, 14408C>T, 22477C>T, 23403A>G, and 25563G>T) among Pakistani viruses of clade GH compared with the reference Wuhan strain (NC_045512). In addition, strain NIH-45090 showed 28027G>T & 29696C>T, NIH-45143 displayed 10738T>A, 25706T>C & 29868G>A and NIH-45579 had 1613C>T, 3613T>C, 18603T>C, 18788C>T, 28378G>C & 28878G>A nucleotide changes. The Pakistani S clade strains contained nine common SNPs (2461T>C, 8782C>T, 11230G>T, 24051A>C, 26313C>T, 28144T>C, 28167G>A, 28878G>A, and 29742G>A). The strain NIH-44905 also had a nucleotide change at position 355C>T (Table 3). When compared with SARS-CoV-2 isolates submitted in GISAID from Pakistan at the same nucleotide positions, three strains UN-UVAS-Sialkot/2020 (EPI_ISL_548946), UN-UVAS-Lahore-I/2020 (EPI_ISL_548942) and KPK-KUST-SJTU/2020 (EPI_ISL_513925) showed same nucleotide variations at position 241C>T, 3037C>T, 14408C>T (ORF1ab) and 23403A>G (S-gene) compared to current study strains of GH clade (NIH-45090, NIH-45143, & NIH-45579). The KPK-KUST-SJTU/2020 contained nucleotide changes in ORF1ab (2416C>T, 8371G>T, 11083G>T), S-gene (22477C>T) and ORF3a (25563G>T). Furthermore, Gilgit1/2020 (EPI_ISL_417444) strain had similar variations at 241C>T and 11083G>T in ORF1ab while KP-RMI-01/2020 (EPI_ISL_632908) showed a single change (11083G>T in ORF1ab) (Fig 3A).

**Table 3:** Mutations in SARS-CoV-2 sequences of Pakistan.

Genome Change	Gene	Protein Change	Mutation Type	Clade
241C>T	5’UTR		Non-coding	GH
1613C>T	ORF1ab	L450F	Missense	
2416C>T			Synonymous	
3037C>T			Synonymous	
3613T>C			Synonymous	
8371G>T		Q2702H	Missense	
10738T>A		N3491K	Missense	
11083G>T		L3606F	Missense	
14408C>T		P4715L	Missense	
18603T>C			Synonymous	
18788C>T		T6175I	Missense	
22477C>T	S		Synonymous	
23403A>G		D614G	Missense	
25563G>T	ORF3a	Q57H	Missense	
25706T>C		F105S	Missense	
28027G>T	ORF8	W45L	Missense	
28378G>C	N		Synonymous	
28878G>A		S202N	Missense	
29696C>T	3’UTR		Non-coding	
29868G>A	3’UTR		Non-coding	
355C>T	ORF1ab		Synonymous	S
2461T>C			Synonymous	
8782C>T			Synonymous	
11230G>T	ORF1ab	M3655I	Missense	
24051A>C	S	D830A	Missense	
26313C>T	E		Synonymous	
28144T>C	ORF8	L84S	Missense	
28167G>A		E92K	Missense	
28878G>A	N	S202N	Missense	
29742G>A	3’UTR		Non-coding	

The amino acid (a.a) changes between SARS-CoV-2 strains in the current study and the reference virus NC_045512 were also determined. Overall, eleven missense mutations were found among the Pakistani strains of clade GH. Five missense mutations were common in all the strains, three in ORF1ab polyprotein (a.a Q2702H, L3606F, P4715L), one in surface glycoprotein (a.a D614G), and one in ORF3a protein (a.a Q57H). Furthermore, strain NIH-45090 showed a missense mutation in ORF8 protein (a.a W45L), strain NIH-45143 revealed novel amino acid substitutions in ORF1ab polyprotein (a.a N3491K) and in ORF3a protein (a.a F105S). Strain NIH-45579 showed two amino acid changes in ORF1ab polyprotein (novel mutation a.a L450F and T6175I), and one in nucleocapsid protein (a.a S202N). The two Pakistani strains of clade S contained five missense mutations: one in ORF1ab polyprotein (a.a M3655I), a novel mutation in surface glycoprotein (a.a D830A), two missense mutations in ORF8 protein (a.a L84S and E92K), and one in nucleocapsid phosphoprotein (a.a S202N) (Table 3). The comparison of amino acid changes between the current study strains with available sequences from Pakistan was also carried out. The KP-RMI-01/2020 (EPI_ISL_632908) and Gilgit1/2020 (EPI_ISL_417444) strains showed a similar amino acid change (L3606F in ORF1ab) with our GH clade strains. Pakistani strains UN-UVAS-Sialkot/2020 (EPI_ISL_548946) and UN-UVAS-Lahore-I/2020 (EPI_ISL_548942) contained one amino acid change each in ORF1ab (a.a P4715L) and surface glycoprotein (a.a D614G), respectively. Moreover, five mutations were found in KPK-KUST-SJTU/2020 (EPI_ISL_513925) i.e. three in ORF1ab (a.a Q2702H, L3606F & P4715L), one each in surface glycoprotein (a.a D614G) and OFR3a (a.a Q57H) (Fig 3B).

## Discussion

As of December 25, 2020, the 22,269 whole-genome sequences of SARS-CoV-2 available on the GISAID from Asia showed that the majority of these strains belong to the GR clade, followed by G and GH. In the present study, three strains (NIH-45090, NIH-45143, and NIH-45579) belong to the GH clade, while two strains (NIH-HAS001 and NIH-44905) clustered in S clade which is very rare in Asia. The global distribution of these clades between January-December 2020 showed that GH was found at low frequency during the start of the pandemic (January-February 2020). However, increased frequencies were reported in March (21%), April (26%), and May (30%). The GH clade strains peaked in June (31%) in agreement with our findings and showed constant presence during July-October 2020. A second rise in infections due to GH clade was observed during November-December 2020 (35% and 45% respectively). On the contrary, S clade was dominant during January-February 2020 (42% and 30% respectively) and since then has shown a rapid decline and was reported with a frequency of 1% in November 2020 (https://www.gisaid.org/epiflu-applications/phylodynamics/).

According to the Nextstrain classification and phylogenetic analysis, Pakistani strains (NIH-45090, NIH-45143, and NIH-45579) of 20A clade were closely related to the strains from Oman, Slovakia, United States, and indigenous strain EPI_ISL_513925. Similarly, the Pakistani strains of 19B clade were closely related to the strains from India and Oman. We assume multiple introductions of SARS-CoV-2 strains into Pakistan particularly through international travelers. Eight independent events of importation were observed (based on the analysis of available sequence data from Pakistan) mainly from the neighboring countries especially Oman where a large proportion of Pakistani people are settled and employed for years. These findings suggest that the transmission of SARS-CoV-2 in Pakistan was influenced by very frequent and recurrent introductions through international travelers that lead to the circulation of different clades over time. Although the interventions at a national scale such as bans on indoor and outdoor gatherings may help to restrict and combat the community transmission chains, the frequent introductions through international travelers have also impacted the transmission dynamics. The limited sample size and genomic information available warrants further epidemiological and genomic surveillance for the more accurate estimation of SARS-CoV-2 diversity and transmission behaviors across the country.

Spike protein of SARS-CoV-2 is considered a major target for drug and vaccine design because of its involvement in—recognition of host cell receptor, attachment, and entry [21, 22]. Notably, we have found a D614G (aspartic acid to glycine) mutation in the envelope spike protein of indigenous strains (NIH-45090, NIH-45143, and NIH-45579). Moreover, the D614G change was accompanied by three characteristic mutations: 241C>T, 3037C>T, and 14408C>T. The haplotype containing these four mutations has now become the most prevalent form globally; before March, the haplotype was found in 10% of global sequences but increased substantially to 67% during March and 78% between April-May 2020. During the initial outbreak in China, nearly all the strains had aspartic acid at position 614; however, later strains with glycine 614 emerged in Europe, followed by North America, Oceania, and Asia [4]. Recently, Korber et al., reported that the G614 form is more infectious, and infected patients had a higher upper respiratory tract viral load; however, association with disease severity was not observed [4]. *In vitro* studies conducted by Li et al., involving pseudoviruses showed that the D614G mutation was responsible for increased infectivity [23]. Similar findings have been reported where the G614 mutant showed the highest cell entry among the spike variants [7] as well as efficient infection of 293T-ACE2 cells compared to D614 pseudovirus [6]. Moreover, experimental work by Hou et al., demonstrated that the D614G substitution enhances SARS-CoV-2 infectivity, competitive fitness, and transmission in primary human cells and animal models [24]. In another study, an increased case fatality rate reported in European countries and the East Coast of the United States was correlated with the increased prevalence of the G614 variant [25]. In Pakistan, an upsurge in COVID-19 cases (n = 141,010 cases out of total 213,470; 66%) and deaths (n = 2,852 deaths out of total 4,395; 64.8%) were reported during the month of June 2020 compared with those reported during February-May 2020 (72,460 confirmed cases and 1,543 deaths). This substantial increase in numbers of cases and deaths may indicate widespread circulation of the G614 variant, which needs to be further investigated [4, 25]. Although the D614G mutation is increasingly reported in strains worldwide, we have found a novel mutation D830A (aspartic acid to alanine) in strains NIH-44905 and NIH-HAS001 of clade S. Additional molecular epidemiological studies are needed to monitor the circulation of the G614 and A830 variant strains in Pakistan, which might also help to clarify the impact of SARS-CoV-2 genetic variants that might affect disease severity and clinical management. Furthermore, tracking mutations in spike glycoprotein of SARS-CoV-2 is important due to its role in host cell receptor binding and entry as well as eliciting neutralizing antibodies. Since the detection and global spread of the G614 variant, a major concern has been on the impact of D614G mutation on the effectiveness of vaccines as most of the vaccines have been designed using the D614 virus (Lurie et al., 2020). This concern has been addressed by the findings of Weissman et al., which showed that the variant with D614G mutation does not escape neutralization but rather is neutralized at a higher level by serum from vaccinated mice, non-human primates, and humans [26]. However, continuous surveillance of genetic variations in spike protein of SARS-CoV-2 is imperative to detect any escape mutants and for future vaccine development programs.

In summary, we report 29 mutations in the genomic sequences of Pakistani SARS-CoV-2 strains of GH and S clades. Both the clades showed missense mutations in the spike gene; however, clade S had less overall variability compared with the Wuhan reference strain than the GH clade strains. This highlights the fact that circulating SARS-CoV-2 strains in Pakistan have evolved and have sequence variations compared with the early ancestors. Therefore, we recommend whole-genome sequencing of indigenous strains prevalent in different regions of the country to understand virus evolution and to identify strains with unique mutational patterns.

## Supporting information

S1 File (TXT)

S1 Data (XLSX)

## References

[1] World Health Organization. Weekly Operational Update on COVID-19, October 16, 2020. https://www.who.int/emergencies/diseases/novel-coronavirus-2019/situation-reports.

[2] Pakistan confirms first two cases of coronavirus. https://www.france24.com/en/20200226-pakistan-confirms-first-two-cases-of-coronavirus.

[3] Tai W, He L, Zhang X, Pu J, Voronin D, et al. (2020) Characterization of the receptor-binding domain (RBD) of 2019 novel coronavirus: implication for development of RBD protein as a viral attachment inhibitor and vaccine. Cellular & molecular immunology 17: 613–620. 10.1038/s41423-020-0400-4 32203189 PMC7091888

[4] Korber B, Fischer WM, Gnanakaran S, Yoon H, Theiler J, et al. (2020) Tracking changes in SARS-CoV-2 Spike: evidence that D614G increases infectivity of the COVID-19 virus. Cell 182: 812–827. e819. 10.1016/j.cell.2020.06.043 32697968 PMC7332439

[5] Zhang L, Jackson CB, Mou H, Ojha A, Peng H, et al. (2020) SARS-CoV-2 spike-protein D614G mutation increases virion spike density and infectivity. Nature communications 11: 1–9. 10.1038/s41467-019-13993-7 33243994 PMC7693302

[6] Hu J, He CL, Gao Q, Zhang GJ, Cao XX, et al. (2020) The D614G mutation of SARS-CoV-2 spike protein enhances viral infectivity. BioRxiv. 10.1101/2020.06.20.161323

[7] Ozono S, Zhang Y, Ode H, Seng TT, Imai K, et al. (2020) Naturally mutated spike proteins of SARS-CoV-2 variants show differential levels of cell entry. BioRxiv. 10.1101/2020.06.15.151779

[8] Plante JA, Liu Y, Liu J, Xia H, Johnson BA, et al. (2020) Spike mutation D614G alters SARS-CoV-2 fitness. Nature: 1–6. 10.1038/s41586-020-2895-3 PMC8158177 33106671

[9] Yurkovetskiy L, Wang X, Pascal KE, Tomkins-Tinch C, Nyalile TP, et al. (2020) Structural and functional analysis of the D614G SARS-CoV-2 spike protein variant. Cell 183: 739–751. e738. 10.1016/j.cell.2020.09.032 32991842 PMC7492024

[10] Holshue ML, DeBolt C, Lindquist S, Lofy KH, Wiesman J, et al. (2020) First case of 2019 novel coronavirus in the United States. New England Journal of Medicine 5;382(10):929–936. 10.1056/NEJMoa2001191 PMC7092802 32004427

[11] Fraser C, Donnelly C, Cauchemez S, Hanage W, Van Kerkhove M, et al. (2009) WHO Rapid Pandemic Assessment Collaboration. Science 324: 1557. 10.1126/science.1176062 19433588 PMC3735127

[12] Gardy JL, Loman NJ (2018) Towards a genomics-informed, real-time, global pathogen surveillance system. Nature Reviews Genetics 19: 9. 10.1038/nrg.2017.88 29129921 PMC7097748

[13] Fauver JR, Petrone ME, Hodcroft EB, Shioda K, Ehrlich HY, et al. (2020) Coast-to-coast spread of SARS-CoV-2 during the early epidemic in the United States. Cell 181(5):990-996.e5. 10.1016/j.cell.2020.04.021 32386545 PMC7204677

[14] Lu J, du Plessis L, Liu Z, Hill V, Kang M, et al. (2020) Genomic Epidemiology of SARS-CoV-2 in Guangdong Province, China. Cell 181(5):997-1003.e9. 10.1016/j.cell.2020.04.023 32359424 PMC7192124

[15] Deng X, Gu W, Federman S, du Plessis L, Pybus OG, et al. (2020) Genomic surveillance reveals multiple introductions of SARS-CoV-2 into Northern California. Science 369(6503):582–587. 10.1126/science.abb9263 32513865 PMC7286545

[16] Eden J-S, Rockett R, Carter I, Rahman H, De Ligt J, et al. (2020) An emergent clade of SARS-CoV-2 linked to returned travellers from Iran. Virus Evolution 6: veaa027. 10.1093/ve/veaa027 32296544 PMC7147362

[17] Munnink BBO, Nieuwenhuijse DF, Stein M, O’Toole A, Haverkarte M, et al. (2020) Rapid SARS-CoV-2 whole genome sequencing for informed public health decision making in the Netherlands. Nature Medicine 26(11):1802. 10.1038/s41591-020-1128-5 PMC7574672 33082576

[18] Gudbjartsson DF, Helgason A, Jonsson H, Magnusson OT, Melsted P, et al. (2020) Spread of SARS-CoV-2 in the Icelandic population. New England Journal of Medicine 382(24):2302–2315. 10.1056/NEJMoa2006100 PMC7175425 32289214

[19] Jesus JGd, Sacchi C, Candido DdS, Claro IM, Sales FCS, et al. (2020) Importation and early local transmission of COVID-19 in Brazil, 2020. Revista do Instituto de Medicina Tropical de São Paulo 62. 10.1590/S1678-9946202062030 PMC7232955 32401959

[20] Gambaro F, Baidaliuk A, Behillil S, Donati F, Albert M, et al. (2020) Introductions and early spread of SARS-CoV-2 in France. BioRxiv. 10.1101/2020.04.24.059576 32643599 PMC7346363

[21] Ortega JT, Serrano ML, Pujol FH, Rangel HR (2020) Role of changes in SARS-CoV-2 spike protein in the interaction with the human ACE2 receptor: An in silico analysis. EXCLI journal 19: 410. 10.17179/excli2020-1167 32210742 PMC7081066

[22] Liu Z, Xiao X, Wei X, Li J, Yang J, et al. (2020) Composition and divergence of coronavirus spike proteins and host ACE2 receptors predict potential intermediate hosts of SARS‐CoV‐2. Journal of medical virology 92: 595–601. 10.1002/jmv.25726 32100877 PMC7228221

[23] Li Q, Wu J, Nie J, Zhang L, Hao H, et al. (2020) The impact of mutations in SARS-CoV-2 spike on viral infectivity and antigenicity. Cell 182: 1284–1294. e1289. 10.1016/j.cell.2020.07.012 32730807 PMC7366990

[24] Hou YJ, Chiba S, Halfmann P, Ehre C, Kuroda M, et al. (2020) SARS-CoV-2 D614G variant exhibits efficient replication ex vivo and transmission in vivo. Science 370: 1464–1468. 10.1126/science.abe8499 33184236 PMC7775736

[25] Becerra‐Flores M, Cardozo T (2020) SARS‐CoV‐2 viral spike G614 mutation exhibits higher case fatality rate. International Journal of Clinical Practice 74(8):e13525. 10.1111/ijcp.13525 32374903 PMC7267315

[26] Weissman D, Alameh M-G, de Silva T, Collini P, Hornsby H, et al. (2020) D614G spike mutation increases SARS CoV-2 susceptibility to neutralization. Cell host & microbe 29(1):23-31.e4. 10.1016/j.chom.2020.11.012 PMC7707640 33306985

